# SPARC Inhibits Metabolic Plasticity in Ovarian Cancer

**DOI:** 10.3390/cancers10100385

**Published:** 2018-10-16

**Authors:** Christine Naczki, Bincy John, Chirayu Patel, Ashlyn Lafferty, Alia Ghoneum, Hesham Afify, Michael White, Amanda Davis, Guangxu Jin, Steven Kridel, Neveen Said

**Affiliations:** 1 Departments of Cancer Biology, Wake Forest University School of Medicine, Winston Salem, NC 27157, USA; cmcmahan@wakehealth.edu (C.N.); bincyanujohn@gmail.com (B.J.); cpatel@wakehealth.edu (C.P.); Laffak14@wfu.edu (A.L.); aghoneum@wakehealth.edu (A.G.); hafify@wakehealth.edu (H.A.); michawhi@wakehealth.edu (M.W.); amadavis@wakehealth.edu (A.D.); gjin@wakehealth.edu (G.J.); skridel@wakehealth.edu (S.K.); 2 Wake Forest Baptist Comprehensive Cancer Center, Winston Salem, NC 27157, USA; 3 Departments of Pathology, Wake Forest University School of Medicine, Winston Salem, NC 27157, USA; 4 Departments of Urology, Wake Forest University School of Medicine, Winston Salem, NC 27157, USA

**Keywords:** SPARC, ovarian cancer, syngeneic model, peritoneal metastasis, metabolic plasticity, glycolysis, OXPHOS, redox homeostasis

## Abstract

The tropism of ovarian cancer (OvCa) to the peritoneal cavity is implicated in widespread dissemination, suboptimal surgery, and poor prognosis. This tropism is influenced by stromal factors that are not only critical for the oncogenic and metastatic cascades, but also in the modulation of cancer cell metabolic plasticity to fulfill their high energy demands. In this respect, we investigated the role of Secreted Protein Acidic and Rich in Cysteine (SPARC) in metabolic plasticity of OvCa. We used a syngeneic model of OvCa in *Sparc*-deficient and proficient mice to gain comprehensive insight into the paracrine effect of stromal-SPARC in metabolic programming of OvCa in the peritoneal milieu. Metabolomic and transcriptomic profiling of micro-dissected syngeneic peritoneal tumors revealed that the absence of stromal-*Sparc* led to significant upregulation of the enzymes involved in glycolysis, TCA cycle, and mitochondrial electron transport chain (ETC), and their metabolic intermediates. Absence of stromal-*Sparc* increased reactive oxygen species and perturbed redox homeostasis. Recombinant SPARC exerted a dose-dependent inhibitory effect on glycolysis, mitochondrial respiration, ATP production and ROS generation. Comparative analysis with human tumors revealed that SPARC-regulated ETC-signature inversely correlated with SPARC transcripts. Targeting mitochondrial ETC by phenformin treatment of tumor-bearing *Sparc*-deficient and proficient mice mitigated the effect of SPARC-deficiency and significantly reduced tumor burden, ROS, and oxidative tissue damage in syngeneic tumors. In summary, our findings provide novel insights into the role of SPARC in regulating metabolic plasticity and bioenergetics in OvCa, and shines light on its potential therapeutic efficacy.

## 1. Introduction

The tropism of ovarian cancer (OvCa) to the peritoneal cavity is implicated in widespread dissemination (peritoneal ovarian carcinomatosis, POC), suboptimal surgery, chemo-resistance, and poor prognosis [1,2]. Because of this tropism, OvCa cells exhibit increased fatty acid uptake and depend primarily on fatty acid oxidation for energy generation [3,4]. However, recent studies reported upregulation of glycolytic enzymes in OvCa and the sensitivity OvCa cells to inhibitors of glycolysis and biguanides that target mitochondrial bioenergetics [5,6], suggesting OvCa cell metabolic plasticity, as they depend on multiple metabolic pathways to meet the increasing demands of energy production and building biomass [7,8]. This plasticity is initiated and maintained not only by intrinsic cancer cell factors but also by micro-environmental cues, and has been correlated with chemo-resistance and recurrence [5,7].

We have reported on the tumor suppressor effect of SPARC in OvCa as both stromal- and tumor-derived SPARC inhibits tumor growth and invasiveness, and are implicated in the normalization of the peritoneal tumor microenvironment (TME) [9]. Mechanistic studies using multiple preclinical model systems indicated that SPARC exerts a multi-faceted paracrine effect not only inhibiting OvCa cell proliferation but also inhibits their interactions with stromal factors within the peritoneal milieu inhibiting the invasion and metastasis cascades [9,10,11,12]. Therefore, we sought to investigate the effect of stromal-SPARC on OvCa metabolic plasticity. Herein, we describe a previously unknown function of SPARC suppressing OvCa cell metabolic plasticity through the inhibition of glycolysis, and mitochondrial bioenergetics. These findings thus provide insight into the paracrine effect of SPARC normalizing the OvCa metabolic ecosystem.

## 2. Results

### 2.1. Loss of Stromal-SPARC Is Associated with Enhanced Glycolysis in Syngeneic ID8 Intraperitoneal Tumors

**Figure 1:**
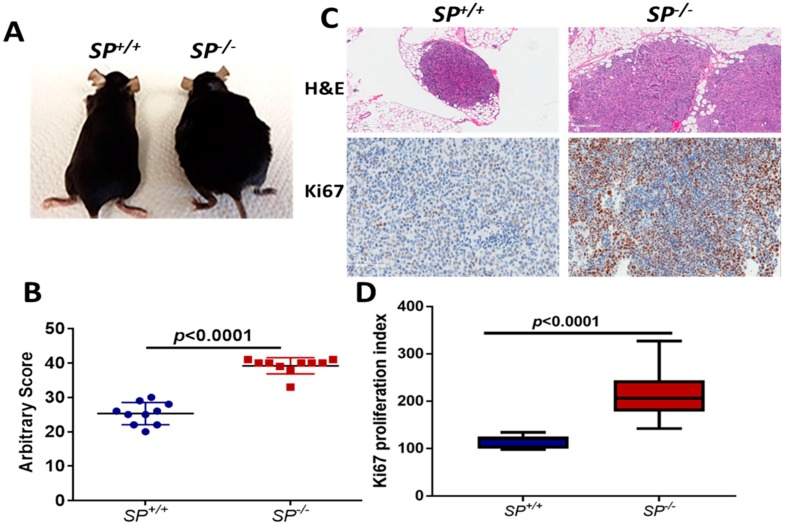
Enhanced growth of ID8 syngeneic tumors in *SP^−/−^* mice. (**A**) photomicrographs of *SP^+/+^* and *SP^−/−^* mice 8 weeks after ip injection of ID8 cells. (**B**) Scatter plots showing the arbitrary scores of tumor burden in *SP^+/+^* and *SP^−/−^* mice. (**C**) H&E staining of ip tumors (100×, upper), Ki67 immunostaining of syngeneic tumor sections (lower, 200×). (**D**) Box plots of the proliferation index determined by counting Ki67 positive nuclei in five random fields/tumor section (n = 3 sections/genotype). *p* values are determined by Mann-Whitney and Student’s *t*-test.

**Figure 2:**
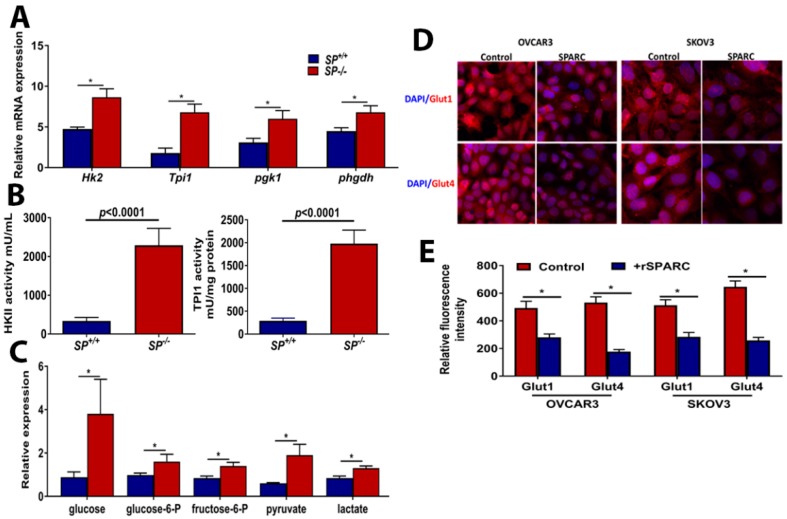
Effect of host-SPARC on glycolysis: (**A**) Bars depicting mean ± SEM of the changes in the transcripts (n = 3/genotype), (**B**) HK2 and TPI1 activity in ID8 tumors grown in *SP^−/−^* and *SP^+/+^* mice. Bars represent the mean ± SEM of the measured (n = 6/genotype; unpaired Student’s *t*-test). (**C**) The levels of glycolysis metabolites (n = 6/genotype) between ID8 tumors grown in *SP^−/−^* and *SP^+/+^* mice. *p* < 0.05, Student’s *t*-test. (**D**) Confocal immunofluorescence showing the expression of glucose transporters Glut1 and Glut4 in OVCAR3 and SKOV3 treated with 10 µg/mL SPARC for 24 h (magnification 100×). (**E**) Bars represent mean ± SEM of the mean fluorescence intensity of expression of Glut1 and Glut4. * *p* < 0.05, Mann-Whitney test.

Syngeneic ID8 OvCa cells in *SP^−/−^* exhibited significantly increased tumor burden with larger omental nodules (Figure 1A–C), and increased proliferation index compared to *SP^+/+^* mice (Figure 1C,D). To investigate whether stromal-SPARC inhibits metabolic programing and plasticity of OvCa, we performed laser captured micro-dissection (LCM) of syngeneic ID8 tumors. Integrated transcriptomic and metabolomic analysis using the Integrated Molecular Pathway Level Analysis (IMPaLA; http://impala.molgen.mpg.de/) [13] indicated significant enrichment of pathways involved in glycolysis, TCA, and oxidative phosphorylation (Supplementary Table S1). Syngeneic tumors from *SP^−/−^* mice exhibited significant upregulation of transcripts of the glycolytic enzymes hexokinase 2 (*Hk2*), triose phosphate isomerase (*Tpi1*), phosphoglycerate kinase (*Pgk1*) and phosphoglycerate dehydrogenase (*Phgdh*) (Figure 2A). The increased transcript levels of *Hk2* and *Tpi1* in ID8 tumors in *SP^−/−^* mice were associated with a significant increase in their enzymatic activity (Figure 2B). To confirm the direct paracrine effect of SPARC, treatment of human and murine OvCa cells with recombinant human and murine SPARC (rSPARC) revealed that rSPARC exhibited dose-dependent inhibition of HK2 and TPI1 activity in human (SKOV3, OVCAR3, CAOV3 and IGROV1) and murine ID8 OvCa cell lines (Supplementary Figure S1). Concurrently, the levels of glucose and glycolysis intermediates glucose-6-phosphate and fructose-6 phosphate were significantly increased in tumors growing in *SP^−/−^* mice compared to the *SP^+/+^*. Consistently, the glycolytic end products pyruvate and lactate were significantly increased (Figure 2C). These data suggest a paracrine effect of SPARC decreasing glucose bioavailability and suppressing glycolysis in syngeneic tumors. We also found that treating OvCa cell lines SKOV3 and OVCAR3 with rSPARC significantly decreased the expression of glucose transporters, Glut 1 and 4 on both cell lines as determined by confocal fluorescence microscopy (Figure 2D,E). Importantly, we found that the levels of glycolytic enzymes, which are upregulated in tumors from *SP^−/−^* mice, are also upregulated in human HGSC compared to normal tissues in studies curated from Oncomine (Supplementary Table S2). 

**Figure 3:**
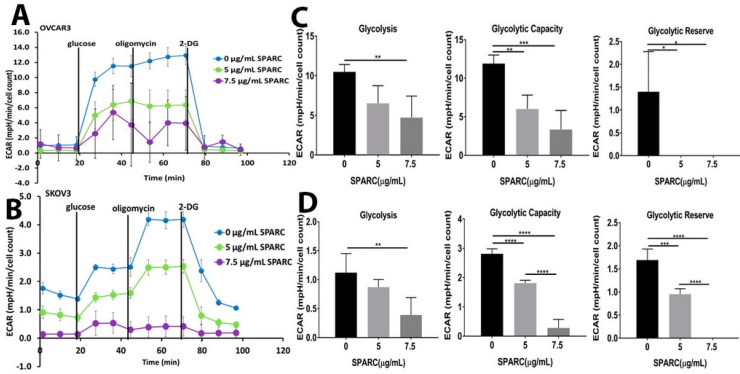
Effect of SPARC on glycolysis: (**A**,**B**) ECAR Seahorse tracing of a representative of three experiments of OVCAR3 and SKOV3 treated with the indicated concentrations of SPARC for 24 h and were subjected to glycolysis stress assay as described in Materials and Methods. (**C**,**D**) Bars represent mean ± SEM of glycolysis, glycolytic capacity and glycolytic reserve of experiments described in (**A**,**B**) (n = 5/experimental condition in each experiment) corrected to the number of viable cells counted by trypan blue exclusion. * *p* < 0.05, ** *p* < 0.01, *** *p* < 0.001, and **** *p* < 0.0001, respectively, one-way ANOVA with multiple comparisons and Tuckey’s post-hoc test.

To investigate whether the enhanced glycolysis in *SP^−/−^* tumors is due to the lack of a paracrine effect of SPARC or due to metabolic adaptation of the rapidly growing tumors in absence of stromal-*Sparc*, we monitored the changes in the kinetics of extracellular acidification rate (ECAR) as a functional readout of glycolysis in human OvCa cell lines in response to rSPARC using Seahorse Agilent platform. We found that SPARC significantly inhibited glycolysis, glycolytic capacity, and glycolytic reserve in both SKOV3 and OVCAR3 in a concentration-dependent manner (Figure 3). Together, these data indicate a direct paracrine effect of SPARC inhibiting glycolysis in OvCa cells in vitro as well as in vivo in syngeneic tumors in *Sparc*-deficient mice.

### 2.2. Effect of Loss of Stromal-SPARC on TCA Cycle and Pentose Phosphate Pathway (PPP)

**Figure 4:**
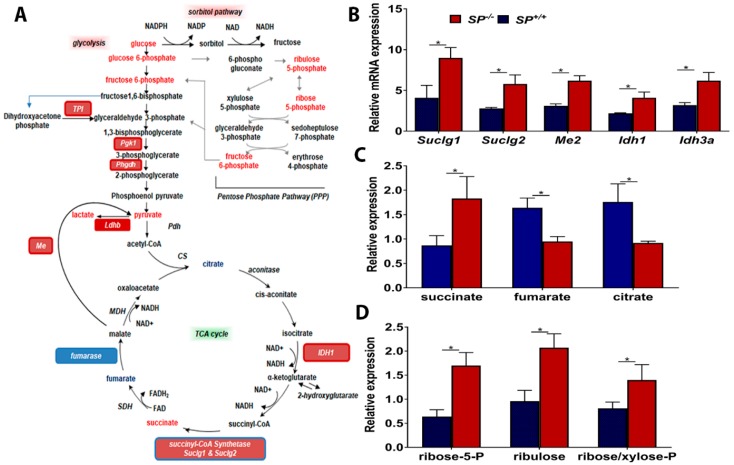
Effect of stromal-SPARC on TCA cycle and pentose phosphate pathway (PPP). (**A**) Schema of glysolysis, TCA and PPP pathways showing the upregulated (Red) and the downregulated (blue) enzymes and metabolites. (**B**) Bar graph representing mean ± SEM of the changes in the transcripts (n = 3/genotype), and (**C**) metabolites (n = 6/genotype) between ID8 tumors grown in *SP^−/−^* and *SP^+/+^* mice. (**D**) Bar graph representing mean ± SEM of metabolites of PPP (n = 6/genotype) in syngeneic tumors. * *p* < 0.05, Student’s *t*-test.

Syngeneic tumors growing in *SP^−/−^* mice exhibited significant upregulation of enzyme transcripts and intermediate metabolites of TCA cycle (Figure 4A–C). The transcripts of isocitrate dehydrogenase isoenzymes (*Idh1* and *Idh3a*) that catalyze the oxidative decarboxylation of isocitrate to alpha-ketoglutarate (α-ketoglutarate) [14] were significantly upregulated. Moreover, *SP^−/−^* tumors exhibited significant upregulation of the transcripts of *Succinyl-CoA synthetase*, a mitochondrial enzyme composed of *succinyl-CoA ligase* subunits alpha and beta (*Suclg1* and *Suclg2,* respectively) [14] that catalyze the reversible formation of succinyl-CoA and succinate and couple the hydrolysis of succinyl-CoA to the synthesis of either ATP or GTP [14]. Consistently, the levels of succinate were significantly upregulated in tumors growing in *SP^−/−^* mice compared to those growing in the *SP^+/+^* (Figure 4B,C). Concomitantly, the levels of fumarate significantly decreased in tumors from *SP^−/−^* mice, suggesting either anaplerotic contributions from other pathways or a disruption in oxidative metabolism. However, citrate was diminished in tumors from *SP^−/−^* mice suggesting either a disruption in pyruvate dehydrogenase function/expression or increased citrate export to the cytoplasm for fatty acid synthesis. Moreover, the transcript level of malic enzyme isoform 2 (*Me2*) was significantly increased. ME2 is one of three isoforms of oxidative decarboxylases, MEs, that catalyze the oxidative decarboxylation of L-malate to pyruvate while simultaneously reducing NAD(P)+ to NAD(P)H [15,16]. ME2 is a mitochondrial NAD(P)+ isoform, and because it affects two cofactors, it plays a key role in physiologic and pathologic functions, including insulin release, rapidly proliferating cancer cells, as well as epithelial-mesenchymal transition (EMT) [15,16,17,18]. Consistent with the transcript level, the enzymatic activity of ME2 in omental nodules from *SP^−/−^* mice is significantly increased as evidenced by a significant increase in its metabolic end product pyruvate (Figure 4). However, the potential function of *ME2* has not been reported in OvCa.

To determine the relevance of our model system to human disease, we found that SPARC-inhibited TCA enzymes are upregulated in human HGSC compared to normal tissues in studies curated from Oncomine (Supplementary Table S2). These TCA enzymes exhibited significant upregulation with the exception of SUCLG1 and IDH3a that exhibited a trend of upregulation though insignificant (Supplementary Table S2). The increased glucose bioavailability in tumors from *Sparc*-deficient mice was also reflected in the increase in the metabolites of sorbitol and pentose phosphate pathways (PPP) as ribose 5-phosphate, ribulose and ribose/xylose-phosphate (Figure 4D). The activation of PPP is crucial for nucleotide synthesis to support cell proliferation and enhanced tumor growth observed in *SP^−/−^* compared to *SP^+/+^* littermates. These results not only indicate the metabolic plasticity of OvCa cells using glycolysis and the TCA cycle for energy production and generation of intermediates for anabolic tumor growth, but also support the paracrine inhibitory effect of SPARC.

### 2.3. Loss of Stromal-Sparc Increases OXPHOS with Overexpression of Mitochondrial ETC Enzymes

**Figure 5:**
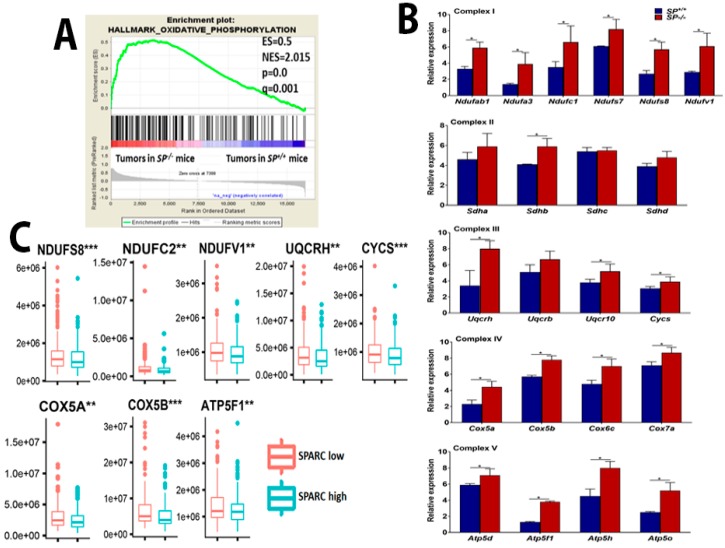
Effect of SPARC on mitochondrial electron transport chain (ETC). (**A**) Gene Set Enrichment Analysis (GSEA) of the top upregulated genes in whole transcriptome of ID8 tumors grown in *SP^−/−^* compared to *SP^+/+^* mice showing the enrichment of oxidative phosphorylation and reactive oxygen species in tumors from *SP^−/−^* mice. (**B**) Bars represent mean ± SEM of the transcript levels of the indicated mitochondrial ETC enzymes determined by qRT-PCR, n = 3/genotype each in triplicates. * *p* < 0.05, Student’s *t*-test. (**C**) Human HGSC data sets from TCGA were stratified based on SPARC transcript level as SPARC-low and SPARC-High. The levels of the indicated enzymes were compared between SPARC-low and SPARC-High groups as described in the material and methods. ** *p* < 0.01 and *** *p* < 0.001.

We next investigated whether the rapid growth of ID8 tumors in *SP^−/−^* mice is associated with upregulation of oxidative phosphorylation to generate more energy. Gene Set Enrichment Analysis (GSEA) indicated selective enrichment of oxidative phosphorylation in tumors growing in *SP^−/−^* compared to *SP^+/+^* mice (Figure 5A). Transcripts of electron transport chain (ETC) enzymes, complex I (NADH dehydrogenase), III (coenzyme Q-cytochrome C reductases), IV (cytochrome C oxidases), and V (ATP generating) were significantly upregulated in tumors from *SP^−/−^* compared to *SP^+/+^* mice (Figure 5B). However, there was no significant difference in the expression of complex II (succinate dehydrogenases) between tumors growing in the two genotypes. Comparative analysis with human HGSC revealed that many of these enzymes are significantly upregulated in SPARC-low compared to SPARC-high tumors (Figure 5C). We also found that many of these enzymes were also upregulated in human HGSC compared to normal tissue studies curated in oncomine (Supplementary Table S2). Upregulation of these enzymes was also reported in advanced OvCa and in OvCa stem cells with invasive and metastatic potential [15]. These data indicate that in the absence of stromal-*Sparc*, OvCa cells upregulate and utilize ETC and OXPHOS to generate more energy together with glycolysis and the TCA cycle to fulfill their high demands of energy and metabolites.

**Figure 6:**
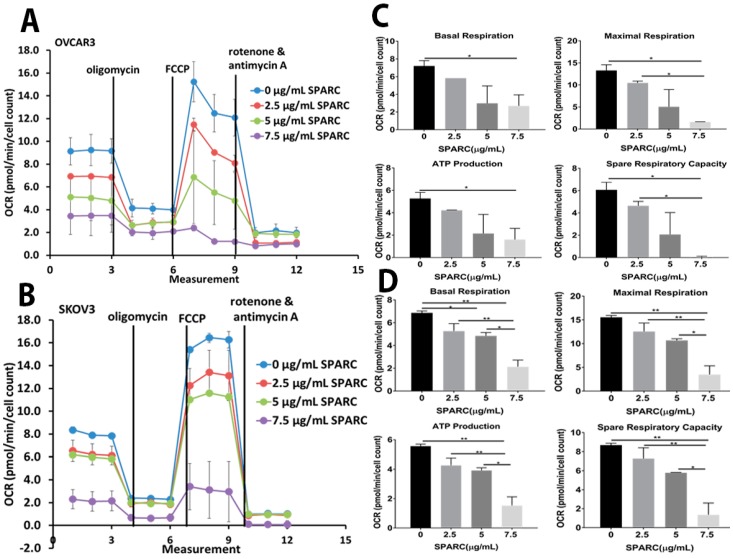
Effect of SPARC on mitochondrial functions and bioenergetics. (**A**,**B**) Seahorse tracing of the oxygen consumption rate in OVCAR3 and SKOV3 treated with the indicated concentrations of SPARC for 24 h, followed by mitochondrial stress test as described in material and methods. (**C**,**D**) Bar graphs of means ± SEM of the basal and maximal respiration, ATP production and spare respiratory in OVCAR3 and SKOV3 cells treated with SPARC recorded in a representative of three experiments (Five replica/experimental condition/experiment). * p < 0.05, ** p < 0.01, one-way ANOVA with Tuckey’s post-hoc test.

Given that mitochondria are the central hub for cellular metabolic pathways, we next investigated the direct paracrine effect of SPARC on mitochondrial functions. We monitored oxygen consumption rate (OCR) in SKOV3 and OVCAR3 treated with increasing doses of rSPARC (Figure 6A,B). In addition, we found that SPARC exerted dose-dependent inhibition of basal and maximal respiration, spare respiratory capacity and spare respiratory reserve. Importantly, SPARC significantly inhibited ATP production from both cell lines in a dose-dependent manner (Figure 6C,D).

### 2.4. Effect of SPARC on Mitochondrial Functions

**Figure 7:**
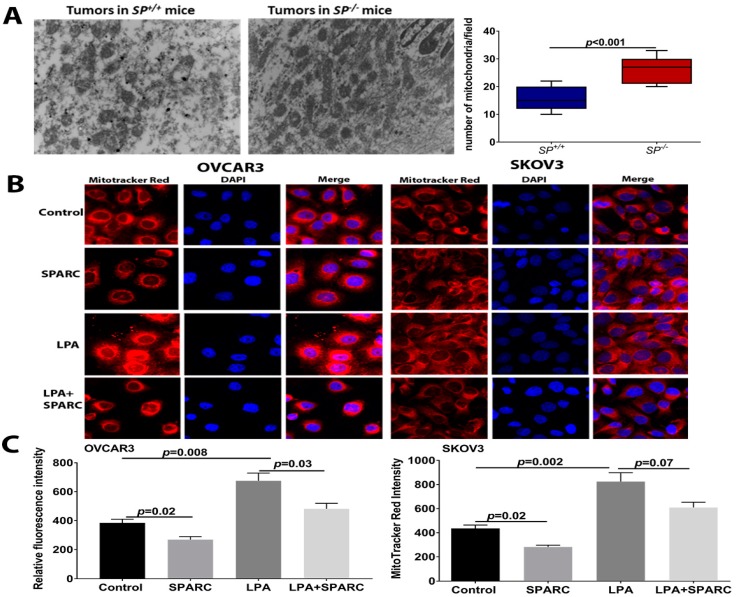
Effect of SPARC on mitochondrial mass: (**A**) Electron microscopic images of the mitochondria in ID8 syngeneic tumors in *SP^−/−^* and *SP^+/+^* mice (top). Bars depict the mean ± SEM of the number of mitochondria in five independent fields/section, n = 4/genotype. *p* < 0.05, Student’s *t*-test. (**B**) MitoTracker red fluorescent staining of respiring mitochondria in OVCAR3 and SKOV3 treated with LPA in the presence and absence of SPARC. (**C**) Bars depict the relative fluorescent intensity of Mitotracker stain. *p* < 0.05, Mann-Whitney Test.

To investigate whether the altered metabolic profiles (described above) in *the absence of stromal-SPARC* are due to abnormal mitochondrial function, we examined the number of mitochondria in syngeneic tumors that grew in both genotypes. Electron microscopic examination of tumor sections revealed a significantly increased number of mitochondria in the *SP^−/−^* tumors compared to the *SP^+/+^* (Figure 7A). In tandem, treatment of OvCa cells with SPARC significantly decreased the mass of respiring mitochondria as determined by MitoTracker red fluorescence (Figure 7B,C). Interestingly, stimulation of OvCa cell lines with LPA, a bone a fide OvCa promoting factor, resulted in a twofold increase in mitochondrial mass. Co-treatment with rSPARC significantly decreased LPA-induced mitochondrial mass (Figure 7B,C).

### 2.5. Perturbed Redox Homeostasis in Tumors Dissected from SP^−/−^ Mice

**Figure 8:**
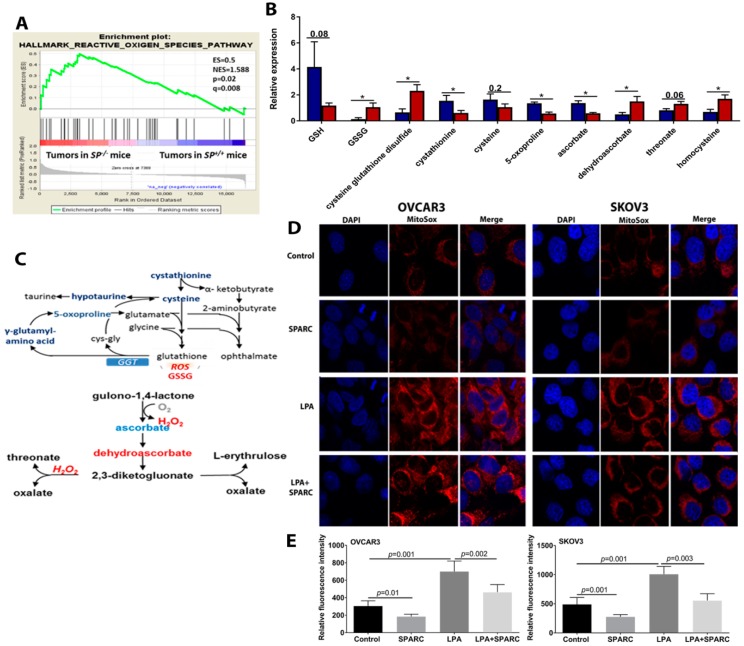
Effect of SPARC on mitochondrial ROS in OvCa cells. (**A**) GSEA graph depicting enrichment of ROS in syngeneic tumors from *SP^−/−^* mice. (**B**) Bars depict the relative expression of the indicated metabolites in syngeneic ID8 tumors in *SP^+/+^* and *SP^−/−^* mice. * *p* < 0.05 unpaired Student’s *t*-test. (**C**) Schema of the pathway of the metabolites involved in redox signaling. Red: upregulated; Blue: downregulated. (**D**) MitoSox fluorescence microscopy of SKOV3 and OVCAR3 treated with LPA with and without SPARC. (**E**) Bars depict mean ± SEM of the fluorescent intensity of images in (**D**) *p* < 0.05, Mann-Whitney’s test.

In addition to ATP generation, mitochondrial OXPHOS is a major cellular source of reactive oxygen species (ROS), mainly H_2_O_2_ from complex I, II and III [16]. Our earlier reports showed the paracrine effect of SPARC inhibiting ROS generation and accumulation of markers of oxidative tissue damage in syngeneic ID8 peritoneal tumors [12] and in carcinogen-induced urothelial cancer in *SP^−/−^* mice compared to the *SP^+/+^* [17]. Consistently, ID8 tumors in *SP^−/−^* mice exhibited significant enrichment of ROS signature (Figure 8A), evidenced by significantly increased accumulation of oxidized glutathione (GSSG) accompanied by reduced levels of glutathione (GSH) indicating disruption in redox homeostasis in the absence of stromal-*Sparc* (Figure 8B). Upon exposure to high levels of reactivate oxygen species (ROS), cysteine oxidation initiates the generation of a disulfide bond with another oxidized molecule of reduced GSH, forming GSSG [18]. In this respect, high levels of cysteine-glutathione disulfide serve as an internal marker of oxidative stress in *SP^−/−^* tissues. Consistently, diminished levels of the antioxidant ascorbate were found in *SP^−/−^* tissues accompanied by the accumulation of the ascorbate oxidation products dehydroascorbate and threonate (Figure 8B,C). *SP^−/−^* tissues also possessed reduced levels of cystathionine and cysteine (the rate limiting metabolites for GSH synthesis) [18], as well as hypotaurine indicating cysteine depletion to support glutathione biogenesis (Figure 8B,C). Collectively, these observations indicate that the enhanced metabolic pathways upregulated in tumors growing in *SP^−/−^* mice lead to increased generation of ROS and perturbed redox homeostasis compared to *SP^+/+^* counterparts.

Because the mitochondria are the central hub for cellular metabolic processes and generation of ROS, we determined the effect of rSPARC on mitochondrial ROS generation using MitoSox fluorescent tracker that specifically detects mitochondrial ROS. We found that the treatment of OvCa cell lines with rSPARC significantly inhibited basal and LPA-induced mitochondrial ROS (Figure 8D). Together, these data indicate that SPARC inhibits mitochondrial bioenergetics, through the downregulation of ETC enzymes, ATP production and ROS generation.

### 2.6. Targeting Mitochondrial ETC by Phenformin Mitigates the Accelerated Growth of OvCa Syngeneic Tumors

**Figure 9:**
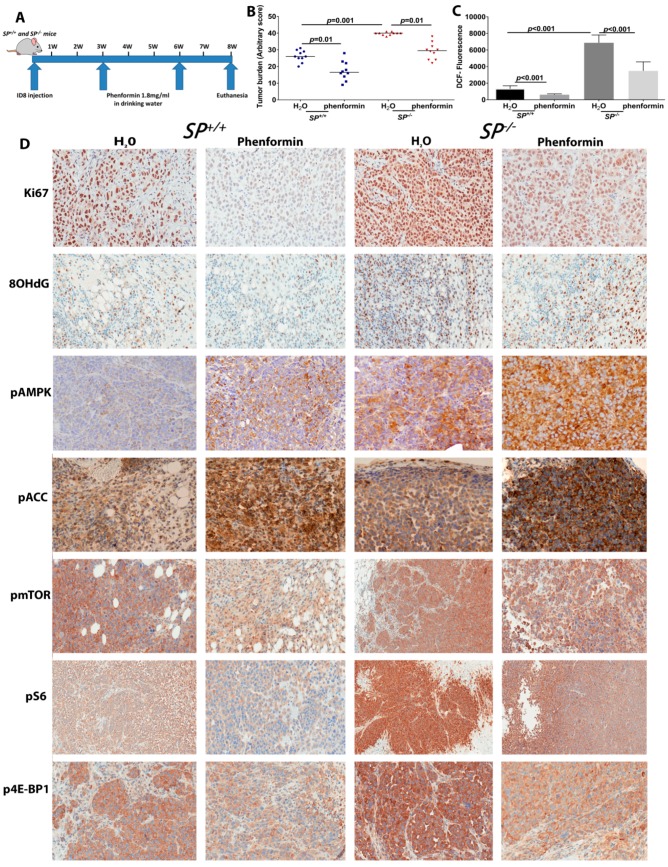
Effect of phenformin on syngeneic ID8 tumors in *SP^−/−^* and *SP^+/+^* mice. (**A**) Schema of the mouse therapeutic trial with phenformin. (**B**) Scatter plots showing the effect of phenformin on syngeneic tumor burden in *SP^−/−^* and *SP^+/+^* mice. *p* < 0.05. (**C**) Bars depict mean ± SEM of the levels of H_2_O_2_ in syngeneic tumors determined by DCF fluorescence. *p* < 0.05, Student’s *t*-test. (**D**) Immunostaining of syngeneic tumors with the indicated antibodies (200×).

Pharmacologic targeting of mitochondrial-dependent metabolism has recently gained intense interest through repurposing the effective anti-type II diabetes biguanides, metformin and phenformin, that inhibit mitochondrial complex I and consequently inhibit the biosynthetic and redox functions of mitochondria [6,19]. Phenformin is a more potent inhibitor of mitochondrial complex I, with broader tissue bioavailability independent of the Organic Cation Transporter 1 (OCT1) [20] and a more effective anti-tumor effect [21]. Thus, we reasoned whether phenformin will mitigate the accelerated tumor growth in *Sparc*-deficient mice. We treated tumor-bearing *Sparc*-deficient and -proficient mice with oral phenformin (1.8 mg/mL in drinking water) [21], starting 3 weeks after ID8 cell injection for a further 2 weeks (Figure 9A). We found that oral phenformin significantly reduced the tumor burden in *SP^−/−^* mice compared to *SP^+/+^* mice, partially mitigating the effect of loss of stromal-*Sparc* (Figure 9B). Phenformin treatment significantly reduced ROS in tumor tissues as determined by DCF fluorescent tracker (Figure 9C). Tumors from phenformin-treated mice exhibited significantly reduced proliferation with decreased nuclear Ki67 immunostaining, compared to untreated controls. Importantly, phenformin significantly decreased proliferation in tumors growing in *SP^−/−^* (Figure 9D and Supplementary Figure S2). Consistent with increased ROS in tumors growing in *SP^−/−^* mice, we found a significant increase of 8-hydroxy-2-deoxy guanosine (8-OHdG) immunostaining a marker of oxidative DNA damage compared to those in the *SP^+/+^*. Altogether, these data indicate that the inhibition of mitochondrial ETC by phenformin significantly mitigates the increased tumor burden, proliferation index, ROS generation and oxidative DNA damage observed in syngeneic tumor growing in absence of stromal-*Sparc*.

Earlier studies reported that the inhibitory effect of phenformin on OvCa is mediated through the activation of adenosine monophosphate kinase (AMPK) and the inhibition of the mechanistic/mammalian target of rapamycin C1 (mTORC1) [22]. In addition, mTORC1 was reported to stimulate mitochondrial activity and biogenesis by selectively promoting the translation of nuclear-encoded mitochondrial ETC enzymes [23] that we found upregulated in the absence of stromal-*Sparc* in our study (Figure 2). The effect of mTORC1 was mediated through phosphorylation of the eukaryotic translation initiation factor 4E (eIF4E)-binding proteins (4E-BPs) [23]. Therefore, we sought to determine whether the inhibitory effect of phenformin described above is mediated through these pathways. Immunostaining of syngeneic tumors revealed that phenformin treatment induced phosphorylation of AMPKα (pAMPK, T^172/183^) in tumors grown in *SP^−/−^* and *SP^+/+^* mice compared to untreated controls (Figure 9D and Supplementary Figure S2). Tumors from untreated *SP^−/−^* showed a modest increase, though insignificant in pAMPK compared to tumors in *SP^+/+^* mice. Phenformin treatment induced significantly more phosphorylation of AMPK in tumors from *SP^−/−^* mice compared to those from the *SP^+/+^*. Consequently, there was more phosphorylation of acetyl CoA carboxylase (pACC) in *SP^−/−^* tumors. These findings support the effect of phenformin inducing the phosphorylation of AMPK and its downstream ACC. However, their increased phosphorylation in absence of stromal-*Sparc* suggests either they augmented the stimulation due to increased energy demands or minimal or there is no implication in the tumor suppressor effect of SPARC in the model system reported herein. On the other hand, tumors from untreated *SP^−/−^* mice exhibited significantly increased expression of phosphorylated mTOR (pmTOR S^2448^) and its downstream targets phospho-p70S6K^T89^ and 4EBP1^T37/46^. Phenformin treatment significantly decreased the expression of pmTOR, p70S6K and p4EBP1 in tumors from *SP^−/−^* mice (Figure 9D and Supplementary Figure S2). These data further indicate that SPARC inhibits OvCa growth through the inhibition of bioenergetics and energy-generating metabolic pathways.

## 3. Discussion

In the present study, we report a novel role of SPARC as a tumor suppressor in OvCa. We used a syngeneic mouse model of disseminated peritoneal OvCa that phenocopies late stages III and IV of human disease known as peritoneal ovarian carcinomatosis. Herein, we report that the accelerated growth of ID8 cells injected in *SP^−/−^* mice is associated with transcriptomic signature suggestive of metabolic plasticity of syngeneic ovarian tumors. This metabolic plasticity is exemplified by significant upregulation of transcripts and metabolites of multiple interconnected metabolic pathways to support the increasing demands of the accelerated tumor growth. Comparative analysis with human data further supported the finding that SPARC-regulated signature is upregulated in advanced human high grade serous cancer (HGSC).

Tumors growing in *SP^−/−^* mice exhibited enhanced bio-energetic pathways including glycolysis, and TCA cycles with significant upregulation of the key enzymes and metabolites associated with the malignant phenotype in many cancers [24,25,26,27,28,29,30,31]; however, the role of these enzymes and metabolites in OvCa is still unraveled. Accelerated glucose metabolism under aerobic conditions, described by Otto Warburg, is one of the hallmarks of cancer [32]. Here we report that in the absence of stromal-SPARC, glucose levels are increased in cancer cells with the concomitant upregulation of *Hk2*, the mitochondrial-associated high affinity hexokinase that catalyzes the first committed step in glucose metabolism, i.e., the ATP-dependent phosphorylation of glucose to glucose-6-phosphate (G6P) [33]. *HK2* is expressed in higher levels in cancer cells and is responsible for the accelerated glucose flux [33]—a property that distinguishes cancer from normal cells and was exploited for non-invasive visualization of cancer cells using ^18^F-fluorodeoxyglucose and positron emission tomography, FDG-PET [33]. Our findings indicate that the loss of SPARC supports interconnected metabolic plasticity of cancer cells feeding metabolites between pathways for the anabolic growth of tumor cells. This was evidenced by the upregulation of *TPI1* that connects glycolysis, gluconeogenesis; while *PHGDH* catalyzes the reaction which diverts 3-phosphoglycerate from glycolysis to the serine synthesis pathway for de novo serine synthesis [34]. Paradoxically, we show the direct paracrine effect of SPARC downregulating the expression of glucose transporters Glut1 and Glut4 and inhibiting glycolysis in human OvCa cell lines.

We report that the loss of stromal-*Sparc* accelerated the TCA cycle at multiple levels. In addition to increased pyruvate generated from glycolysis, *IDH* and *SUCLG* isoenzymes were upregulated driving the forward reactions of the TCA cycle and activating the first energy generating step from succinyl CoA to succinate. These findings provide evidence of the suppressive paracrine role of stromal-SPARC on the interconnected glycolysis and TCA cycle. These were further supported by our findings of the upregulation of the ETC enzymes and the significant increase in the mitochondrial number in tumors growing in absence of stromal-SPARC in vivo as well as the direct paracrine effect of SPARC mitochondrial respiration and oxidative metabolism in vitro. These findings are in agreement with the recent reports of metabolic plasticity and heterogeneity in the metabolic preferences within a given tumor that mitochondrial oxidation of multiple nutrients co-exists with an enhanced glycolytic pathway [35]. However, this plasticity has not been reported in OvCa before.

In the present study, we also report that enhanced cancer cell metabolism in absence of stromal-*Sparc* is associated with increased ROS production and markers of oxidative DNA damage. This is in accord with our earlier findings that the loss of stromal-*Sparc* is associated with increased markers of oxidative tissue DNA, protein and lipid damage [9,12,17]. Altered redox balance subsequently deregulates redox signaling and has been strongly implicated in malignant progression through the stimulation of multiple signaling pathways to promote cancer cell proliferation, invasiveness, angiogenesis and inflammation [36]. Accordingly, cancer cell metabolic plasticity allows cancer cells to adapt to the persistently high endogenous and exogenous ROS levels and enables them to use high ROS and oxidative by-products as well as the cell anti-oxidant machinery as secondary messengers to further promote their growth and metastasis [36].

Interestingly, our findings show a unique phenotypic difference between syngeneic tumors growing in *Sparc*-deficient mice; that is, the significant increase in mitochondrial number may reflect increased mitochondrial biogenesis concomitant with the upregulation of bioenergetic metabolic pathways. This is in accord with earlier studies that reported increased mitochondrial replication with changes in shape, size and number to influence metabolic remodeling of a myriad of physiological and pathological conditions including cancer [37].

Epidemiological studies revealed that the biguanide metformin offers lower overall risk of cancer in diabetic patients compared to those receiving other medications [19,38]. The anti-tumor efficacy of metformin and phenformin promoted clinical trials investigating the use of metformin in cancers including OvCa are currently ongoing (http://www.clinicaltrials.gov). Recent reports highlighted that phenformin is a more potent anticancer agent, with broad-spectrum tissue bioavailability [21] in many cancers including OvCa [22,39]. Our findings that phenformin mitigated the increased tumor burden of syngeneic tumors in *Sparc*-deficient mice suggest that the tumor suppressor effect of SPARC in OvCa is mediated, in part, through metabolic programing of cancer cells targeting the mitochondrial ETC. Together, these data provide evidence that stromal-*Sparc* suppresses tumor growth through paracrine inhibition of multiple interconnected metabolic bioenergetic pathways. This was further corroborated by demonstrating the direct effect of SPARC protein inhibiting these metabolic and bioenergetic pathways in human OvCa cell lines in vitro. Moreover, we demonstrated that the absence of stromal-*Sparc* leads to overexpression of phosphorylated mTORC1 and its downstream targets, p70S6K and 4E-BP1, that play a crucial role in regulation of the metabolic programing of cancer cells and the translation of mitochondrial ETC. We also showed that targeting mitochondrial ETC with phenformin mitigated the increased tumor burden, ROS and oxidative tissue damage that developed in the absence of stromal-*Sparc*. Importantly, phenformin inhibited the basal and increased expression of phosphorylated mTOR-p70S6K-4EBP1 in the absence of stromal-*Sparc*.

Indeed, most of the biological functions of SPARC molecule were deduced from the phenotype of *Sparc*-deficient mice in a plethora of physiological and pathological conditions. Using the syngeneic tumor model system in *Sparc*-deficient mice provided a useful tool not only for phenotypic characterization but also shined the light on novel roles of SPARC as tumor suppressor in OvCa.

## 4. Material and Methods

### 4.1. Cell Culture and Reagents

Murine ID8 cell line was earlier described [9,10,11,40]. Human OvCa cell lines SKOV3, OVCAR3 and CAOV3 (originally from ATCC) were obtained as fresh early passage vials from the Cell and Viral Vector Core Lab (CVVL) at Wake Forest Baptist Medical Center (WFBMC) and Comprehensive Cancer Center. Cell lines were maintained in appropriate cell culture media and were regularly confirmed to be *Mycoplasma*-free by WFBMC-CVVL with no cross-species contamination.

### 4.2. In Vivo Syngeneic Model

*SP^+/+^* and *SP^−/−^* mice are maintained on a C57BL/6 background for at least 10 backcrosses. Mice were housed in a specific pathogen-free (SPF) facility. All animal experiments were approved by IACUCs of the University of Virginia and Wake Forest University Schools of Medicine. ID8 intraperitoneal (ip) injections in *SP^+/+^* and *SP^−/−^* mice was previously described [9,10,11,40,41]. Briefly, ID8 cells (4 × 10^6^/100 µL sterile PBS) were injected intra-peritoneally (ip) in *SP^+/+^* and *SP^−/−^* mice [10]. Tumor growth was monitored twice/week by measurement of mouse weight and girth for 8 weeks at the end of which, mice were euthanized and the intra-peritoneal tumor burden was evaluated using the sum of three assigned arbitrary scores. Tumor growth was monitored twice/week by measurement of mouse weight and girth for 8 weeks at the end of which, mice were euthanized and the ip tumor burden was evaluated using fluorescent stereomicroscope microscope (Leica M205, Buffalo Grove, IL, USA) in the omentum, mesentery, liver, and diaphragm using the sum of three arbitrary scores based on tumor size, number and ascitic fluid volume. Based on tumor size, each animal was assigned a score of 0 to +4, where 0 is tumor-free, +1 indicates nodules <1 mm in diameter, 2+ indicates nodules 1 to 5 mm, 3+ indicates nodules 6–10 mm, and 4+ indicates nodules >1 cm. The second score is based on tumor number/organ, where 0 is tumor-free, +1 indicates 1–5 nodules/organ, 2+ indicates 5–10 nodules/organ, 3+ indicates 10–15 nodules, and 4+ for >20 nodules including the sub-diaphragmatic plaques and omental caking. The third score is based on ascitic fluid volume, where 0 indicates no ascites, 1+ is for volumes <100 µL, 2+ is for 101–200 µL, 3+ is for 200–500 µL, and 4+ is for volumes >500 µL. Dissected tumor tissues were either snap frozen in liquid nitrogen then stored at −80 °C till used or fixed in 10% neutral zinc formalin and embedded in paraffin.

### 4.3. Mouse Therapeutic Trial with Phenformin

Pharmacological grade phenformin was purchased from SelleckChemical, Inc. (Houston, TX, USA). Three weeks after ip ID8 injection, tumor-bearing mice *SP^+/+^* and *SP^−/−^* received vehicle (water), or phenformin (1.8 mg/mL) ad lib in drinking water and their daily intake of fluids was monitored. Fresh phenformin was administered every other day. Treatments went on for 3 weeks, through which mice were monitored, as described above, every other day. Mice were euthanized after 2 more weeks, and tissues were harvested and processed as earlier described.

### 4.4. Immunohistochemistry

Immunostaining of formalin fixed paraffin-embedded mouse tumor tissues was performed as earlier described [17]. Antibodies against Ki67 (clone# TEC-3), 8OH-dG, phospho-mTOR (S2448), phospho-AMPKα-1,2 (T172, T183; ThermoFisher), phospho (p)-ACC (S79P), phospho-4E-BP1 (T37/46), p70S6 Kinase/S6K (T89) were from Dako Cytomation (Santa Clara, CA, USA), Abcam (Cambridge, MA, USA), ThermoFisher (Waltham, MA, USA), Cell signaling Technologies (Danvers, MA, USA), and NovusBio (Littleton, CO, USA) respectively. Appropriate HRP-conjugated secondary antibodies and Diaminobenzidine (DAB) were used (Vector Laboratories, Inc., Burlingame, CA, USA).

### 4.5. Laser Captured Microdissection (LCM)

Formalin-fixed paraffin-embedded (FFPE) tumors (n = 3/group) were cut into 10 µm thickness sections of non-charged glass slides. Sections were de-paraffinized and stained with Arcturus Histogene according to the manufacturer’s instructions. Slides were dehydrated and were allowed to dry before laser capture microdissection (ArcturusXT Laser Capture Microdissection (LCM) System, ThermoFisher). RNA was purified from 1–4 sections using the RNeasy FFPE Kit (Qiagen, Germantown, MD, USA).

### 4.6. RNA Extraction, Amplification, Labeling and Analysis

Fifty ng of total RNA from each tissue sample was amplified using the WT-Ovation FFPE RNA Amplification System (NuGEN Technologies Inc., San Carlos, CA, USA) and purified according to the manufacturer’s recommendations. The FL-Ovation cDNA Biotin Module V2 (NuGEN Technologies Inc.) was then used to prepare fragmented and labeled cDNA targets for GeneChip array analysis using a GeneChip^®^ Mouse Gene 2.0 ST Array (Affymetrix, Santa Clara, CA, USA), hybridized and scanned on a GeneChip scanner at the University of Virginia Genomic Core. Raw signal-intensity data were normalized and log2 transformed using the *simpleaffy* package from Bioconductor Analysis of log intensity distributions, pair-wise correlation, and internal Affymetrix controls were performed to evaluate and ensure data quality [42,43]. For each microarray probe set, the mean log fold change between the replica of each genotype was computed and used to calculate the *SP^−/−^/SP^+/+^* expression. A rank-ordered list was then uploaded to the Gene Set Enrichment Analysis (GSEA) (http://software.broadinstitute.org/gsea/index.jsp), and compared against the Molecular Signatures Database (MSigDB) [44].

### 4.7. Quantitative RT-PCR Analysis

The relative expression of each target gene was calculated using the 2^−ΔΔCT^ method to calculate the average CT value of the housekeeping gene for a single reference gene value. Predesigned murine ribosomal 18S gene was used as a control. Primer sequences are listed in Supplementary Table S3. CFX BioRad software was used in quantitative RT-PCR analyses.

### 4.8. Metabolomic Profiling Sample Preparation

Snap-frozen dissected omental tumor nodules that developed in *SP^+/+^* and *SP^−/−^* mice (n = 6/cohort) were prepared as previously described [45,46,47,48,49,50]. Briefly, samples were de-proteinized and protein-associated metabolites were extracted. The resulting extracts were analyzed by ultra-performance liquid chromatography and mass spectroscopy (UPLC-MS/MS) with positive and negative ion mode electrospray ionization, as well as gas chromatography and mass spectroscopy (GC-MS). Samples were analyzed on a Thermo-Finnigan Trace DSQ fast-scanning single-quadrupole mass spectrometer using electron impact ionization (EI) and operated at unit mass resolving power [45,46,47,48,49,50]. 

### 4.9. Data Extraction, Compound Identification, Metabolite Quantification and Data Normalization

Raw data was extracted, peak identified, QC processed and normalized using proprietary Metabolon’s hardware and software. Compounds were identified by comparison to library entries of purified standards or recurrent unknown entities [45,46,47,48,49,50].

### 4.10. Intracellular ROS Formation in Tumor Tissues

Snap frozen tumor tissues were lysed in RIPA buffer and then 50 μL of tissue lysate or hydrogen peroxide standard were incubated with equal volume of 10 µM DCF in 96-well plates suitable for fluorescence measurement. The mixture was incubated at room temperature for 15–45 min protected from light. H_2_O_2_ was detected with a fluorescence plate reader at 480 nm excitation/530 nm emission.

### 4.11. Total ATP Measurement, Hexokinase 2 and TPI1 Enzyme Activity Assays

Total ATP levels in human OvCa cells were measured by using ATP fluorescent assay (Abcam). Determination of the enzyme activity in tumor tissues and cell lines were carried using HK2 and TPI1 activity assay kits (BioVision Inc., Milipitas, CA, USA) according the manufacturer’s instructions.

### 4.12. Transmission Electron Microscopy (TEM)

Tumor tissue sections were processed for TEM at Wake Forest Baptist Medical Center (WFBMC) Imaging Core Facility according to standard protocols [51]. Briefly, FFPE sections were de-paraffinized and fixed with 2.5% glutaraldehyde in 0.1 M Millonig’s phosphate buffer pH 7.3 for 1 h. Subsequently, the samples were washed 3× in Millonig’s buffer and post-fixed with 1% osmium tetroxide in phosphate buffer for 1 h. After 3× washes in buffer, the samples were dehydrated through ascending series of ethanol followed by resin infiltration in propylene oxide. Finally, samples were gradually infiltrated with Spurr’s resin and were allowed to cure in a 70 °C oven overnight followed by sectioning 90 nm sections with a Reichert-Jung Ultracut E ultramicrotome, and staining with lead citrate and uranyl acetate. Sections were viewed with an FEI Tecnai Spirit TEM operating at 80 kV and images were acquired with an AMT 2Vu CCD camera.

### 4.13. Glycolysis and Mitochondrial Stress Assays

Extracellular acidification rates (ECAR) and oxygen consumption rates (OCR) were measured using the Agilent Seahorse XF-24 Extracellular Flux Analyzer (Agilent, Santa Clara, CA, USA). Cells were plated at 3 × 10^4^ cells/well in RPMI-1640 with 10% FBS in 24-well plates (Five wells/experimental condition with one blank well in each row). Cells were treated with indicated concentrations of SPARC (Peprotech, Rocky Hill, NJ, USA) for 24 h. ECAR and OCR measurements were carried out. Prior to starting the assay, cells were washed and incubated in Seahorse assay medium (Agilent Seahorse XF DMEM Medium pH 7.4 #103575-100) and supplemented with 25 mM glucose and L-glutamine in a 37 °C incubator without CO2 for 60 min. For ECAR measurement, three compounds were sequentially injected, glucose was supplied to feed glycolysis, followed by oligomycin (ATPase inhibitor, 1 µM), and 2-deoxyglucose (2-DG, glycolysis inhibitor). The following parameters were measured: Glycolysis is the measure of ECAR (mpH/min) reached (maximal ECAR rate) by a given cell after the addition of saturating amounts of glucose. The glycolytic capacity was measured by the maximum ECAR rate reached by a cell after the addition of oligomycin, effectively shutting down oxidative phosphorylation and driving the cell to use glycolysis to its maximum capacity. After 2-DG injection, the glycolytic reserve was calculated by the difference between glycolytic capacity and glycolysis. The glycolytic reserve is a measure of the capability of a cell to respond to an energetic demand as well as how close the glycolytic function is to the cell’s theoretical maximum.

For OCR measurement, the assay medium was supplemented with 25 mM glucose, 6 mM glutamine and 1 mM pyruvate. Oligomycin (1 µM), fluoro-carbonyl cyanide phenylhydrazone (FCCP, 0.2 µM), an ionophore which shuttles H^+^ ions, and rotenone/antimycin A (0.5 µM), which inhibit complex I and III, were sequentially injected where indicated and OCR (pMoles O_2_/min) was measured in real time. First, baseline cellular oxygen consumption (basal respiration) was measured. After oligomycin, OCR depicts ATP-linked respiration (mitochondrial ATP production) by subtracting the oligomycin rate from baseline cellular OCR. FCCP collapses the inner membrane gradient, driving the ETC to function at maximal rate, and the maximal respiration is derived by subtracting non-mitochondrial respiration from the FCCP OCR. Finally, antimycin A/rotenone were added to inhibit ETC function, indicating the non-mitochondrial respiration. The mitochondrial respiratory reserve capacity was calculated by subtracting basal from maximal respiration. Data were normalized to the number of viable cells determined at the end of each experiment by trypan blue exclusion and cell counting. Data were calculated and graphs were plotted using Agilent Seahorse Wave Desktop software and report generator, MS Excel and GraphPad Prism.

### 4.14. Measurement of Mitochondrial Contents

OvCa cells were seeded in 8-well LabTek slide chambers and were stimulated overnight with SPARC (10 µg/mL), LPA (10 µM) or both. After fixing the cells in 4% paraformaldehyde, cells were stained with MitoTracker Red (that measures respiring mitochondria), and MitoSOX (for mitochondrial ROS) according to the manufacturer’s instructions (Molecular Probes). Image acquisition was done using Leica AF6000 Modular System confocal microscope (Leica). Image acquisition, deconvolution, and maximum projection analysis were performed with the program LAS AF (Leica). Morphometric analysis was measured by Image J.

### 4.15. Statistical Analysis

Unless otherwise stated, statistical differences were determined using Student’s *t* test, Mann-Whitney U test, or One Way Analysis of Variance (ANOVA) tests, with Tuckey’s post-test for multiple comparisons where appropriate. Statistical analyses were conducted using GraphPad Prism software. Results were deemed significant at *p* ≤ 0.05.

### 4.16. Bioinformatics Analyses

Gene expression and clinical information of OvCa patients were downloaded from the Genomic Data Commons (GDC, https://portal.gdc.cancer.gov/). The upper quartile of normalized fragments per kilobase of transcript per million mapped reads (FPKM) data of 376 patients’ RNA-seq data were utilized for differential analysis for SPARC. The SPARC-high patients were defined as such because their SPARC gene expressions were higher than the median expression value of all patients, whereas the SPARC-low patients are those with lower expression levels than the median expression value. The genes for the metabolic enzymes were tested for differential gene expression analysis by Student *t*-test.

## 5. Conclusions

Our study shined the light on novel paracrine functions of SPARC-suppressing OvCa through metabolic programming and plasticity of cancer cells. The validation of the paracrine effects of SPARC using recombinant protein with human and murine OvCa cell lines highlighted the role of SPARC as a potential therapeutic candidate that inhibits metabolic reprograming of OvCa cells. This programing represents tumor vulnerability that can be exploited in developing therapeutic strategies. To the best our knowledge, this is the first study to characterize the paracrine inhibitory effect of SPARC in OvCa.

## Supplementary Materials

The following are available online at http://www.mdpi.com/2072-6694/10/10/385/s1, Figure S1: Effect of SPARC on activity of HK2 and TPI1 in human and murine OvCa cell lines. Ovarian cancer cell lines were treated with increasing concentrations of rSPARC for 24 h and the enzyme activity was determined as described in Materials and Methods. Bars mean ± SEM of a representative experiment that was repeated twice with reproducible results (n = 3/experimental condition/experiment). One-way ANOVA with Tuckey’s post-hoc multiple comparisons’ test, Figure S2: Scores of expression scores of immunostaining of syngeneic tumor tissues in Figure 9D. Box plots of IHC scores of the indicated protein expression in syngeneic tumors before and after phenformin treatment. *p* < 0.05 Mann-Whitney test, Table S1: Pathway Analysis of Overlapping Genes and Metabolites using Integrated Molecular Pathway Level Analysis (IMPaLA), Table S2: Oncomine analysis of mRNA/copy number of SPARC-regulated genes involved in glycolysis, TCA cycle, and oxidative phosphorylation in tumor tissues from patients with HGSC compared to normal tissues in the referenced studies, Table S3: Mouse primer sequences used.
