# Supplementary material for: SPARC Inhibits Metabolic Plasticity in Ovarian Cancer
Source: Cancers (Basel). 2018 Oct 16;10(10):385. doi: 10.3390/cancers10100385 (PMC6209984; doi:10.3390/cancers10100385)
Supplement: Supplementary file 1 [file cancers-10-00385-s001.pdf]

Supplementary material

SPARC Inhibits Metabolic Plasticity in Ovarian Cancer

Christine Naczki, Bincy John, Chirayu Patel, Ashlyn Lafferty, Alia Ghoneum, Hesham Afify, Michael White, Amanda Davis, Guangxu Jin, Steven Kridel and Neveen Said*

Table S1. Pathway Analysis of Overlapping Genes and Metabolites using Integrated Molecular Pathway Level Analysis (IMPaLA).

Pathway Name	Pathway Source	Overlapping Genes	Overlapping Metabolites	P_Joint	Q_Joint
The citric acid (TCA) cycle and respiratory electron transport	Reactome	*SUCLG2; NDUFAB1; ATP5F1; UQCRH; ATP5H; COX5B; NDUFV1; ATP5O; IDH3B*	succinate; fumarate	2.28E-05	0.000801
Respiratory electron transport_ ATP synthesis by chemiosmotic coupling_ and heat production by uncoupling proteins.	Reactome	*NDUFAB1; ATP5F1; ATP5H; COX5B; NDUFV1; ATP5O; UQCRH*		0.000843	0.0129
Oxidative phosphorylation - Homo sapiens (human)	KEGG	*NDUFAB1; ATP5F1; ATP5H; ATP6V1G1; COX5B; NDUFV1; ATP5O; UQCRH*	succinate; fumarate	2.00E-05	0.000746
Electron Transport Chain	Wikipathways	*NDUFAB1; ATP5F1; ATP5H; COX5B; NDUFV1; ATP5O; UQCRH*	Succinate	0.000197	0.00405
Oxidative phosphorylation	Wikipathways	*ATP5F1; ATP5O; NDUFAB1; NDUFV1; ATP5H*		0.00394	0.0443
Formation of ATP by chemiosmotic coupling	Reactome	*ATP5O; ATP5F1; ATP5H*		0.00709	0.0693
superpathway of conversion of glucose to acetyl CoA and entry into the TCA cycle	HumanCyc	*SUCLG2; IDH3B; PGK1; TPI1*	succinate; fumarate	0.00116	0.0158
Citrate cycle (TCA cycle) - Homo sapiens (human)	KEGG	*SUCLG2; IDH3B; ACLY*	succinate; fumarate	0.00101	0.0146
adenosine ribonucleotides de novobiosynthesis	HumanCyc	*ATP5F1; ATP5H; ATP6V1G1*	aspartate; fumarate	0.000751	0.0118
Warburg Effect	SMPDB	*SUCLG2; IDH3B; PGK1*	succinate; glutamate; Fumarate	0.00343	0.0405
TCA Cycle	Wikipathways	*SUCLG2; IDH3B*	succinate; fumarate	0.00421	0.0465

Table S2. Comparison expression of SPARC-regulated metabolic genes between cancer and normal ovarian tissues in studies curated from Oncomine.

Gene	Change	*p* Value	No. of Studies	References
**Glycolysis**				
HK2	increase	0.002	5	[1–5]
TPI1	increase	4.26E-5	5	[1–5]
PGK1	Increase	0.017	8	[1–7]
PHGDH	Increase	0.005	7	[1–7]
**TCA cycle**				
SUCLG1	increase	0.339	7	[1–6]
SUCLG2	Increase	0.006	8	[1–7]
IDH1	Increase	8.57E-15	6	[1–6]
IDH3a	Increase	0.179	5	[1–4,6]
ME2	Increase	8.57E-15	6	[1–6]
**OXPHOS**<br>**Complex I: NADH Dehydrogenases**				
NDUFAB1	Increase	2.41E-5	4	[1,2,4,5]
NDUFA3	Increase	0.18	5	[1–5]
NDUFC2	Increase	0.018	5	[1–5]
NDUFS7	increased	0.917	3	[1,3,4]
NDUFS8	Increase	4.7E-5	7	[1–7]
**Complex II: Succinate Dehydrogenases**				
SDHA	Increase	0.014	7	[1–7]
SDHB	Increase	0.012	7	[1–7]
SDHC	increase	0.026	7	[1–7]
SDHD	increase	0.254	5	[1–5]
Complex III: CoQ Cytochrome C Reductase				
UQCR10	increase	0.012	5	[1–5]
UQCRB	Increase	0.013	6	[1–5,7]
UQCRH	Increase	0.016	6	[1–5,7]
CYCS	increase	0.016	6	[1–5,7]
Complex IV: Cytochrome C Oxidase				
COX5A	Increase	1.58E-5	7	[1–5,7]
COX5B	increase	7.29E-5	7	[1–4,7]
COX6C	increase	0.019	5	[1–4,7]

Table S2. *Cont.*

Gene	Change	*p* Value	No. of Studies	References
Complex V: ATP Synthase				
ATP5F1	increase	1.40E-10	7	[1-5, 7]
ATP5D	increase	0.177	7	[1,2,4–7]
ATP5H	increase	0.482	7	[1–6]
ATP5O	increase	0.092	7	[1–5,7]

Table S3. Mouse primer sequences used.

Accession No.	Primer Name	F 5'–3'	R 5'–3'	Product Size
NR_046235.1	18s rRNA	GTAACCCGTTGAACCCCATT	CCATCCAATCGGTAGTAGCG	151
NM_009415.2	*Tpi1*	GTCAATGATGGGGTGGCTCA	GCAGTGCTCATTGTTTGGCA	169
NC_000072.6	*Hk2*	CGGGAATGGCGTAGATCTGG	GGCCCTCCTTTGTCTGTCTAC	143
NM_001360744.1	*Ndufab1*	CACCCCCACTGACGTTAGAC	GTGAGGAAGAAGGCTCTGATACT	180
NM_025348.2	*Ndufa3*	GGGAGAATCTCTGCCTTCCT	TCATCTCTCACAGGCACTGG	173
NM_016966.3	*Phgdh*	GGAGGCTTTCCAGTTCTGCT	CTGCGATCCCCTCTCCCTAT	108
NM_008811.2	*Pdh*	GCAGAAATTCATCCGTGGTT	CTCCCTTGCCTTTAGCACAG	203
NM_145494.2	*ME2*	TGCGCTCTGCTTTAAGCTTG	TTGGAATTCGGTGAGCACTC	135
NM_001111320.1	*Idh1*	TGGCTGCTTGCATTAAAGGC	TTGGCCTGAGCTAATTTGGC	112
NM_173011.2	*Idh2*	ATGAACATCGGCTCATCGAC	TTTGCAAGCCCACACAAAGC	74
NM_029573.2	*Idh3a*	ACATTGCAGGCAAGGACATG	AAAAGTCCCATGTGGCGAAG	79
NM_008828.3	*pgk1*	TTGAATGGGAAGCCTTTGCC	TGTGTTCCATTTGGCACAGC	128
NM_019879.3	*Suclg1*	TGGCCACATTCACAAGAAGG	TGGGTTGTTTGGTGAACTGC	84
NM_011507.3	*Suclg2*	TGCTGAGTTCCGACAAAAGG	TCACGAAGCAGGCAATGTTC	128
NM_025523.1	*Ndufc1*	TCGACCGTGTTGAAGAGCAG	TCGACCGTGTTGAAGAGCAG	70
NM_133666.3	*Ndufv1*	TTTGGCCGAGAACGCAATTC	TGACACCACCAGCATGTTTC	130
NM_029272.3	*Ndufs7*	TCAAGCGTGAACAGAAGCTG	TCTGGGATTATCGTGCTCAGC	71
NM_001271443.1	*Ndufs8*	TTGCCTGCAAACTCTGTGAG	ATGTCATAGCGTGTCGTTCG	100
NM_025641.3	*Uqcrh*	TACTCTGGTTGCGCTTGTTG	AGCATCTTTCGTTCGTCCTC	70
NM_026219.2	*Uqcrb*	AAGTGGCTGGATGGTTTTCG	AAAGGTCCTCAGGAAGCCTTC	133
NM_007808.4	*Cycs*	TTGGAGTTTTGGGCTGATGG	TTGGCCTTCAAGTGTAGTGC	111
NM_197979.3	*Uqcr10*	GTTTCAAGTTGCCGTTCACC	GCTGACAGCTGTACTCAAACAC	99
NM_007747.2	*Cox5a*	ATTGATGCCTGGGAATTGCG	TCTACATGCTCGCAATGCAG	99
NM_009942.2	*Cox5b*	TGGTTTTGGCTGCACAAAGG	AAGATAACACAGGGGCTCAGTG	103
NM_053071.2	*Cox6c*	TGTTGCCCAAACCACAGATG	AACTTATAGGCAGCGGCAAC	103

NM_009944.3	*Cox7a*	TGCTGAGGACGCAAAATGAG	TTCTCTGCCACACGGTTTTC	101

Table S3. *Cont.*

Accession No.	Primer Name	F 5'–3'	R 5'–3'	Product Size
NM_025313.2	*Atp5d*	ACTTTTCCGCCACCATGTTG	GCAAAGGTGAAGGACATCTGTC	142
NM_009725.4	*Atp5f1*	AGAACATGATGCGTCGCAAG	TGAGCCTTCTTTGCAAGCAG	142
NM_027862.1	*Atp5h*	TCATGCCCCAAAACCAGAAG	AGTCAATCGCAGGTGGTTTC	105
NM_138597.2	*Atp5o*	GCACCGTCAAAGTGAAAAGC	AGGCGACCATTTTCAGCAAG	100

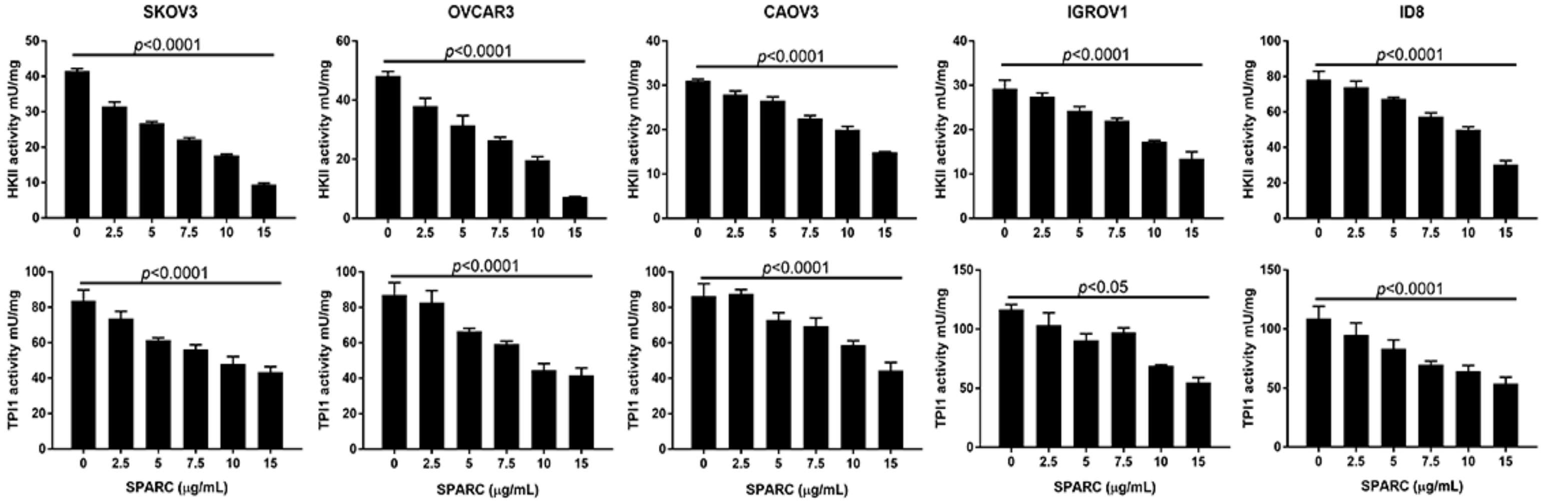

Figure S1. Effect of SPARC on activity of HK2 and TPI1 in human and murine OvCa cell lines. Ovarian cancer cell lines were treated with increasing concentrations of rSPARC for 24 h and the enzyme activity was determined as described in Material and Methods. Bars mean ± SEM of a representative experiment that was repeated twice with reproducible results (n = 3/experimental condition/experiment., One-way ANOVA with Tuckey's post-hoc multiple comparisons' test.

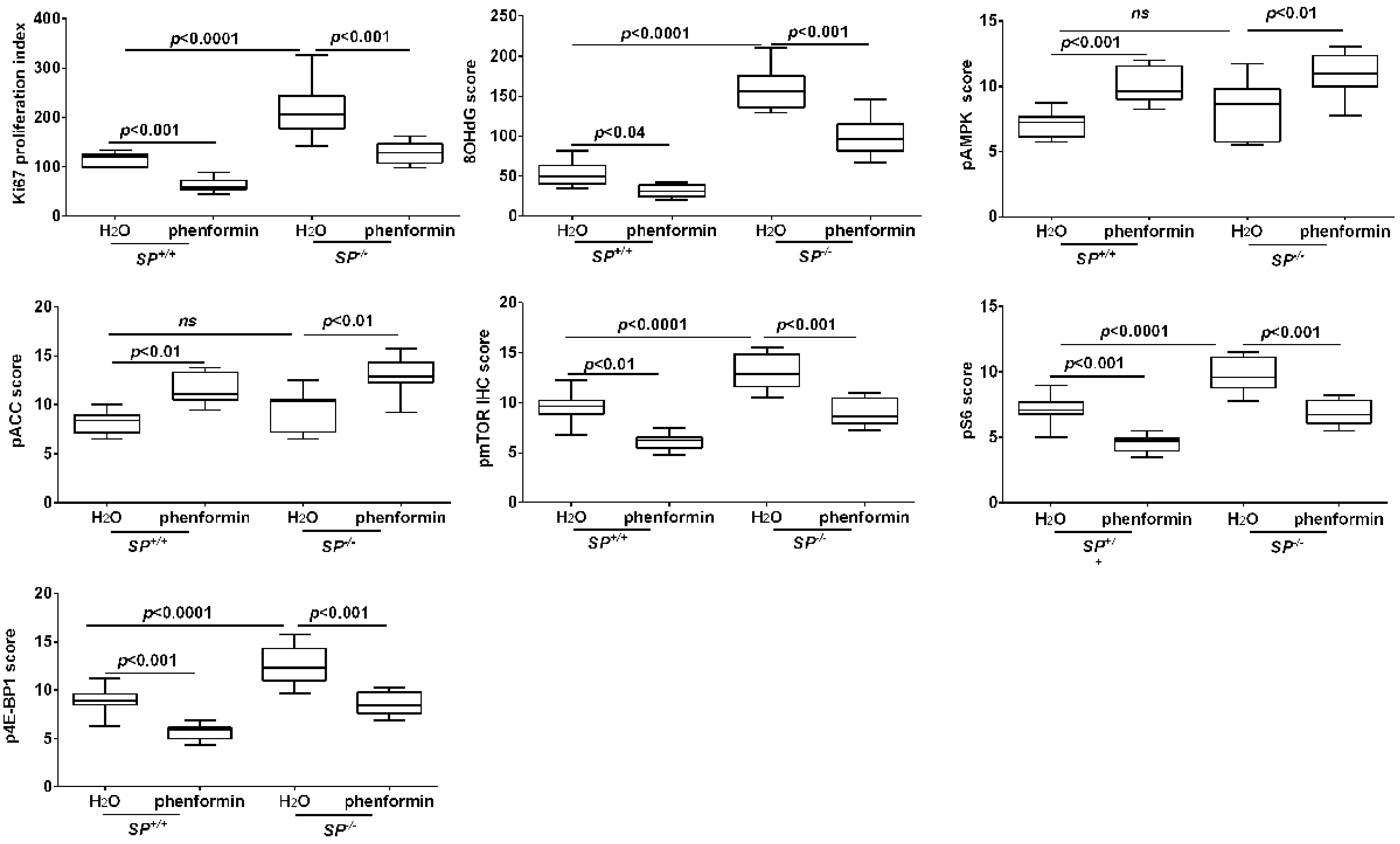

Figure S2. Scores of expression scores of immunostaining of syngeneic tumor tissues in Figure 9D. Box plots of IHC scores of the indicated protein expression in syngeneic tumors before and after phenformin treatment. $p < 0.05$ Mann-Whitney test.

References

1. Bonome, T.; Levine, D.A.; Shih, J.; Randonovich, M.; Pise-Masison, C.A.; Bogomolniy, F.; Ozbun, L.; Brady, J.; Barrett, J.C.; Boyd, J.; et al. A gene signature predicting for survival in suboptimally debulked patients with ovarian cancer. *Cancer Res*. **2008**, *68*, 5478–5486.
2. Hendrix, N.D.; Wu, R.; Kuick, R.; Schwartz, D.R.; Fearon, E.R.; Cho, K.R. Fibroblast growth factor 9 has oncogenic activity and is a downstream target of Wnt signaling in ovarian endometrioid adenocarcinomas. *Cancer Res.* **2006**, *66*, 1354–1362.
3. Lu, K.H.; Patterson, A.P.; Wang, L.; Marquez, R.T.; Atkinson, E.N.; Baggerly, K.A.; Ramoth, L.R.; Rosen, D.G.; Liu, J.; Hellstrom, I.; et al. Selection of potential markers for epithelial ovarian cancer with gene expression arrays and recursive descent partition analysis. *Clin. Cancer Res.* **2004**, *10*, 3291–3300.
4. Network, C.G.A.R. Integrated genomic analyses of ovarian carcinoma. *Nature* **2011**, *474*, 609–615.
5. Yoshihara, K.; et al. Gene expression profiling of advanced-stage serous ovarian cancers distinguishes novel subclasses and implicates ZEB2 in tumor progression and prognosis. *Cancer Sci.* **2009**, *100*, 1421–1428.
6. Adib, T.R.; Henderson, S.; Perrett, C.; Hewitt, D.; Bourmpoulia, D.; Ledermann, J.; Boshoff, C. Predicting biomarkers for ovarian cancer using gene-expression microarrays. *Br. J. Cancer* **2004**, *90*, 686–692.
7. Welsh, J.B., Zarrinkar, P.P.; Sapinoso, L.M.; Kern, S.G.; Behling, C.A.; Monk, B.J.; Lockhart, D.J.; Burger, R.A.; Hampton, G.M. Analysis of gene expression profiles in normal and neoplastic ovarian tissue samples identifies candidate molecular markers of epithelial ovarian cancer. *Proc. Natl. Acad. Sci. USA* **2001**, *98*, 1176–1181.
